# Method for Malaria Diagnosis Based on Extractions of Samples Using Non-Invasive Techniques: An Opportunity for the Nursing Clinical Practice

**DOI:** 10.3390/ijerph17155551

**Published:** 2020-07-31

**Authors:** Adela Gómez-Luque, Juan Carlos Parejo, Maria Zoraida Clavijo-Chamorro, Fidel López-Espuela, Faustin Munyaruguru, Silvia Belinchón Lorenzo, Isabel Monroy, Luis Carlos Gómez-Nieto

**Affiliations:** 1 Department of Nursing, Nursing and Occupational Therapy College, University of Extremadura s/n, 10003 Cáceres, Spain; adelagl@unex.es (A.G.-L.); zoraidacc@unex.es (M.Z.C.-C.); 2 Unidad de Genética, Facultad de Veterinaria, University of Extremadura s/n, 10003 Cáceres, Spain; jucapar@unex.es; 3 Nemba Hospital, BP.15 Ruhengeri, 2055 Rwanda, Africa; rufastin@yahoo.fr; 4 Laboratorio LeishmanCeres, Unidad de Parasitología, Facultad de Veterinaria, University of Extremadura s/n, 10003 Cáceres, Spain; sibelo@unex.es (S.B.L.); isamonry@unex.es (I.M.); cgomez@unex.es (L.C.G.-N.)

**Keywords:** malaria, *Plasmodium falciparum*, DNA, blood specimen collection, noninvasive techniques

## Abstract

Malaria has been for millennia one of the best known and most destructive diseases affecting humans. Its high impact has aroused great interest for the development of new effective and reliable diagnostic techniques. Recently it has been recently published that hairs from mammal hosts are able to capture, hold and finally remove foreign DNA sequences of *Leishmania* parasites. The aim of this study was to check if *Plasmodium falciparum* (*P. falciparum*) DNA remains stable in blood samples deposited in Whatman paper after suffering different transport and storage conditions, and to compare the sensitivity of these results with those offered by thick a smear and Rapid Diagnostic Test, and besides to examine whether *P. falciparum* DNA would be detected and quantified by Real time quantitative PCR (qPCR) from hairs of people with different types of malaria. *P. falciparum* Histidine Repeat Protein II (pHRP-II) antigen detection and *P. falciparum* DNA were detected in 18 of 19 dry blood samples adhered to Whatman paper (94.74%), besides, *Plasmodium* DNA was also detected in seven out of 19 hair samples analyzed (36.84%), remaining stable until analysis for several months under the exposure to different environmental conditions. Although the sensitivity of PCR for the diagnosis of malaria in hair samples is not as high as blood analysis, the study of *Plasmodium* DNA presence in blood and hair could constitute a complementary tool with numerous advantages in sample collection, transport and storage. We suggest that the method could be also applied to medical, forensic and paleo-parasitological diagnosis, not only for malaria but also for searching many other pathogens in hair samples.

## 1. Introduction

Malaria is one of the best known and most destructive disease affecting humans. It is transmitted by the female *Anopheles* mosquito and caused by protozoan parasites of the genus *Plasmodium.* Five species of this genus are known to cause infections in humans—*Plasmodium falciparum*, *Plasmodium vivax*, *Plasmodium malariae*, *Plasmodium ovale* and *Plasmodium knowlesi*, with *P.falciparum* being the most virulent and deadly species.

The 2019 World Health Organization report stated that there were about 228 million cases worldwide, compared with 251 million cases in 2010 and 231 million cases in 2017. Despite deaths from malaria have been reduced from 400,000 in 2010 to 260,000 in 2018, the sub-Saharan area of Africa and India remain the most affected areas in the world [1]. The high morbidity and mortality have led to the launch of several initiatives to eradicate malaria [2], as the development of new effective diagnostic methods that can even detect *Plasmodium* in asymptomatic patients or with unspecific symptoms [3]. 

Currently, diagnostic methods implemented in most surveillance programs include microscopy and rapid diagnostic tests (RDTs), both recommended by WHO [1]. Microscopic diagnosis using blood smears is considered the gold standard, not only for the identification of *Plasmodium* species, but also for the quantification of parasite load in blood. However, detection can be limited in those cases with low parasite loads, in addition to having other drawbacks such as the need for an optical microscope, clean glass slides, immersion oil, reagents for staining, and most importantly, a skilled microscopist [4,5]. 

On the other hand, immunological techniques and the development of various RDTs have allowed quick diagnosis in the field, although with rather high economic costs [6]. However, RDTs have limitations in those cases with low parasite density or due to mutations in diagnostic markers [5]. Considering the decrease of the prevalence in many areas and the low detection limits of microscopy and RDTs, there is the need for the development of effective diagnostic strategies for field application [7].

Molecular methods are a suitable alternative, although costs and technical requirements hamper currently its implementation in areas with limited resources [8]. PCR methods, described by the first time in 1990 [9], are currently improving the diagnosis of malaria, enabling the detection of DNA from different *Plasmodium* species. Although this technique is more expensive than traditional thick smear, it has advantages such as its high precision and sensitivity [10,11,12]. Blood is the chosen sample used for the molecular diagnosis of *Plasmodium* [7], mainly when is blotted to a filter paper. This is an easy and harmless way of sampling due to the small amount of required blood, with significant advantages for the patients and also for the transport and storage of the samples, and it has been commonly used in field studies [13,14]. 

Nowadays, in rural areas of developing countries, the accurate diagnosis based on non-invasive samples is very necessary and demanded, especially in cases where sampling must be repeated [15]. Despite blood is the most used sample for the molecular diagnosis, the presence of *Plasmodium* DNA has been also described in urine and saliva [16,17,18,19]. This fact shows that nowadays there is a great interest in the research of noninvasive procedures for the diagnostic of malaria, using body secretions and the mucosal surface for the detection of parasitic DNA, without harming the patients. Regarding parasitic DNA detection in non-invasive samples, it has been recently published that hairs from mammals such as domestic dogs [20] and wild reservoirs [21], as well as laboratory BALB/c mice [22] are able to capture, hold and finally remove foreign kinetoplast DNA (kDNA) sequences of *Leishmania infantum* and *Leishmania major* parasites, respectively. Dielectrophoretic and magnetophoretic approaches have been recently proposed such as promising new techniques due to their high specificity for red blood cells infected with the malaria parasite [3].

Taking these into account, one of the aims of this study was to check if *Plasmodium falciparum* (*P. falciparum*) DNA remains stable in blood samples deposited in Whatman paper after suffering different transport and storage conditions, and to compare the sensitivity of these results with those offered by thick smear and RDTs. 

On the other hand, we wanted to examine whether *P. falciparum* DNA could be detected and quantified by Real time quantitative PCR (qPCR) from hairs of people with different types of malaria, and if it would be possible to apply this method to diagnose the disease.

We think that this sampling method may help in malaria diagnosis, especially in situations where is difficult to find laboratory facilities, as in rural areas. We consider that countries with greater economic resources must collaborate on the eradication of malaria by detecting asymptomatic patients, involving themselves in the WHO universal access strategy for the malaria diagnosis, and providing the diagnosis in other countries with imported malaria due to migratory movements and international travelling.

## 2. Materials and Methods

### 2.1. Design

A transversal descriptive study was conducted with 19 patients attended in Nemba District Hospital (Rwanda) selected in a consecutive way. All of them came from the district of Gakenke (Rwanda, Africa) with confirmed malaria caused by *P. falciparum* (10 women and 9 men, aged from 4 to 31).

Several types of malaria (uncomplicated (U), severe (S) and cerebral (C)) and different grades of severity and range of symptoms were present in these patients. Malaria was diagnosed by thick blood smear, and parasite load was assessed using the plus system [23], as follows: slight (+, 1–10 parasites/100 thick film fields), moderate (++, 11–100 parasites/100 thick film fields), high (+++,1–10 parasites/thick film field) and very high (++++,>10 parasites/ thick film field).

The remaining volume of blood from routine thick blood smear diagnosis was deposited in two 20 mm diameter circles of 3MM Whatman (Maidstone, UK) filter paper (100 µL of blood/ circle) and dried at room temperature (18–22 °C). Besides, head hairs without roots (about 50 units) were obtained from all patients. Hairs were cut closest to the bulb with sterile scissors. As all patients had short hair (1–4 cm), we obtained approximately 25 cm/hair per patient. All samples were introduced in sterile resealable plastic bags and were properly identified, including the clinical history of each person. 

Samples were shipped to LeishmanCeres laboratory (Spain) and then maintained in different storage conditions until analysis to check the stability of samples: at room temperature (4 months at 18–24 °C), refrigeration (3 months at 2–6 °C) and freezing (1 month at −20 °C and 1 month at −80 °C). 

### 2.2. Analytical Techniques

#### 2.2.1. Rapid Diagnostic Test (RDT)

To confirm the microscopic diagnosis of malaria, a rapid diagnostic test was performed. For this purpose, from each patient 50µL of blood (a half of one paper disk) was incubated in 50µL of PBS + 0.05% Tween-20 for 30 min at 37 °C. Approximately, 5 µL of the supernatant was used to conduct an immunochromatographic analysis using the On Site Pf/Pan Malaria Ag Rapid Test (CTK Biotech Laboratories, San Diego, CA, USA), following the manufacturer’s instructions. This kit detects antibodies against specific *P. falciparum* Histidine Repeat Protein II (pHRP-II), and also against *Plasmodium* Lactate Dehydrogenase (pLDH), a Pan antigen common for the four malaria species (*P. falciparum*, *P. ovale*, *P. malariae* and *P. vivax*). A positive result only for pHRP-II band indicates an uncomplicated infection by *P. falciparum*. Positivity results only for the pLDH band shows the presence of infection caused by other species different from *P. falciparum*. The reaction against the two bands could be interpreted as positive against *P. falciparum*, without discarding the possibility of a mixed infection by other species.

#### 2.2.2. Real Time PCR (qPCR) 

These analyses were conducted to evaluate the sensitivity of blood-qPCR, to compare the results with those obtained by microscopy and with the RDT and also to examine whether *P. falciparum* DNA could be detected and quantified in head hairs. For these purposes, one blood disk and about 25 cm of hair per patient were incubated separately for 2 h at 37 °C in 250 µL (hair) or 500 µL (blood) of lysis buffer (10 mM TrisCl, 0.1 EDTA, 0.5% SDS + 20 µg/mL pancreatic RNase) with 100 µg/µL of proteinase K (Thermo Fisher Scientific, Waltham, MA, USA). After an overnight incubation at 56 °C, DNA from all samples was obtained with Ultra Clean Blood Spin kit (Mo Bio Laboratories, Carlsbad, CA, USA) according to the manufacturer’s instructions. The detection and quantification of parasite DNA were performed using the Primer Design™ Quantification of *P. falciparum* Advanced Kit (Gene Sig Laboratories, Southampton, UK), designed to target a sequence within the plasmeps in 4 genes of *P. falciparum* that has a very low homology with other species of plasmodia that cause malaria. PCR reactions were carried out in 96-well PCR plates in a final volume of 20 µL (5 µL of DNA + 15 µL of Reaction Mix), containing 1 µL of *P. falciparum* Primer/Probe Mix, 1 µL of internal extraction control primer/probe Mix, 3 µL of RNAse/DNAse free water and 10 µL of iTaq^™^ Universal Probes Supermix (Biorad Laboratories, Hercules, CA). The thermal cycling profile was: incubation at 50 °C for 2 min, initial denaturation step at 95 °C for 10 min, 50 cycles of denaturation at 95 °C for 10 s and finally an annealing-extension at 60 °C for 1 min. Positive (*P. falciparum* DNA) and negative (RNAse/DNAse free water) controls and an internal DNA extraction control (used to detect the presence of PCR inhibitors) were included in the assay. All PCR analyses were performed in a Step One Plus Real Time PCR System (Applied Biosystems Laboratories, Foster City, CA, USA). To detect and quantify parasite DNA, a standard curve was carried out using 4 quantities (from 10,000 to 10 copies) of the *P. falciparum* DNA included in the kit, analyzed together with the samples in duplicate. Samples were considered positive when the threshold cycle (Ct) of almost one of the wells was lower than the Y-intercept value (the expected Ct for 1 copy of the target gene).

### 2.3. Ethics

The study was designed according to the Declaration of Helsinki, and the protocol was approved by the Nemba District Hospital ethics committee (REF: 252-16). All subjects were previously informed, they understood the objectives and practices involved in the study and they signed an informed consent document. In terms of minor patients, the collection of samples was done in the presence of their parents or guardians after they have signed the informed consent.

Blood samples were obtained as part of routine clinical procedure for the diagnosis of malaria. Furthermore, at that moment, hair samples were obtained to be studied in this work. These samples were collected by the medical personnel involved in the study, who coded and anonymized them.

## 3. Results

The patients selected for this work came from the same malaria endemic district and showed different manifestations of the disease (uncomplicated, severe and cerebral). All of them were diagnosed as positive to *P. falciparum* by microscopic thick blood smear technique, with different degrees of parasite load (from slight to very high), and some of them received treatment against malaria (Table 1).

The RDT using blood in paper disks after several storage conditions confirmed the presence of *P. falciparum* (positive results in pHRP II band), in 18 out of 19 patients (94.74%) (Table 1), with 12 of them also positive for pan-specific pLDH antigen (63.16%). Patient no. 3, who was treated with quinine and had slight parasite load, showed negative results in both RDT bands. However, RDT was able to detect *P. falciparum* in patient no.12, who also had a slight parasite load, but did not receive treatment. Moreover, positive results for pLDH band alone were not found.

In terms of blood-qPCR analyses, *P. falciparum* DNA was detected in 18 out of 19 patients (94.74%), with copies of the target gene ranging from 11.68 to 171,040.16 (Table 1). The highest no. of DNA copies in blood was observed in patient no. 11, who showed reactivity in both bands of the RDT and had very high parasite load diagnosed by thick blood smear (Table 1). The lowest DNA concentrations were observed in patients with slight or moderate parasite load, except in patients no. 4 (moderate parasite load, 312.47 DNA copies) and no. 13 (very high parasite load, 59.76 DNA copies). Interestingly, patient no. 18, who was negative to blood-qPCR, showed positive results in the RDT pHRP II band, whereas in patient no. 3, who did not show reactivity in RDT, 14.74 DNA copies were detected by qPCR. Both of them were young males with cerebral malaria treated with quinine (Table 1).

In addition, we were able to detect *P. falciparum* DNA in the hair of seven out of 19 patients by qPCR (36.84%) ranging the no. of DNA copies from 3.8 to 177.73 (Table 1). These seven patients were positive to at least one band of the RDT and to blood-qPCR, and presented high or very high parasite load measured by thick blood smear. However, it was not possible to state a correspondence between hair and blood-qPCR results, since high parasite loads detected in blood were not related to positive results in hair samples.

Despite five out of seven hair positive samples belonged to nontreated patients, the highest DNA concentrations in hair (patients no. 2 and 11; 177.73 and 73.77 DNA copies respectively), were found in people with cerebral malaria treated with quinine, who also showed very high parasite load by thick smear (Table 1).

## 4. Discussion

*P. falciparum* and other malaria-causing species are mainly present in Africa and parts of Rwanda [24], the study site. The 19 analyzed clinical cases represented examples of sick children, pregnant women and acute or complicated forms of a parasitic disease that causes thousands of deaths, which occur worldwide every year. The high impact of the disease has generated great interest in the development of new early and reliable diagnostic techniques, essential for the effective management and control of malaria. WHO recommends immediate parasitological confirmation in all suspected cases, before administering treatment [1]. For these reasons, it is important to improve the diagnostic techniques in malarial diseases.

Immune chromatography-based rapid diagnostic tests have been used since 1990, and nowadays are very common in field studies, using blood to diagnose this disease. In our work we used an RDT which has been evaluated by the WHO [25,26], and our results have shown high sensitivity (94.74%) in the detection of infections by *P. falciparum*, except in one patient with low parasitemia. These are similar results to those were reported by other RDTs applied to fresh blood samples that show a sensitivity of around 95% of the cases confirmed by microscopy [27,28,29,30] Pan-specific pLDH detection showed a lower sensitivity, 12 of 19 patients (63.16%), which is slightly higher than that reported in low transmission seasons (58.0%) in Niger [31]. These results could point to the possibility of a mixed infection, but it is highly unlikely according to the epidemiological data offered by the WHO [32], which indicate that the 100% of cases reported in Rwanda in the last year were confirmed as simple *P. falciparum* infections. The Pan-malarial pLDH antigen has been described also as a useful tool to monitor success of antimalarial therapy, as the recognition of the pLDH band turns negative after a successful treatment [31,33]. It should be noticed that, in our case, all the pLDH negative results corresponded to patients who had received treatment against the disease. In our work, the use of dried blood included in filter paper to conduct the RDT, could be an advantage in situations in which fresh blood is not available, such as in the developing countries. In these areas, where malaria is more common, only few hospitals and health centers are available in relation to the population and its dispersion. In addition, the collection of blood samples in filter papers is a good source for molecular diagnosis in malaria [13,14]. The high sensitivity obtained in the analyzed samples (94.74%) indicates that this method could be an alternative for the extraction of blood samples to diagnoses malaria, however, unfortunately, molecular testing cannot, as yet, completely replace microscopic analysis.

However, the quality of the DNA recovered from dried blood spots decreases with storage time [13]. In our work, the DNA obtained from the dried blood disks after different storage conditions and using a commercial DNA extraction kit was enough to detect *P. falciparum* DNA by qPCR in 18 out of 19 patients (94.74%), with confirmed malaria. According to the results, there seems to be a certain correlation between the parasitic load present in blood by microscopy with the another one detected by quantitative PCR, as previously reported [14,34,35].

In relation to malaria diagnosis by qPCR, the presence of *Plasmodium* DNA in other samples besides blood, such as urine and saliva, was recently demonstrated [16,17,18,19]. The recent demonstration of foreign parasitic DNA presence in hair and epidermal keratinocytes of *Leishmania*-infected animals opened a new suitable scientific field to study the physiology of these epithelial cells during several diseases, acting as a very specialized tissue to sequestrate and eliminate foreign genetic material [20,21,22,36,37]. The detection of *P. falciparum* DNA in malaria patients’ hair confirms again the excretion and purification function of this tissue. It also allows the development of a new tool for epidemiological testing, having significant methodological advantages since the samples are obtained by a noninvasive way. In addition, the foreign DNA is highly stable inside the hair [21] due to the hydrophobic nature of the cuticle and the presence of keratin [38].

It is true that there are still many questions which need to be further assessed, about the chronology and mechanism for *Plasmodium* DNA incorporation in the hair of malaria patients. According to the data presented, positive hair samples corresponded to patients with either uncomplicated or cerebral malaria. The negative data, that constitute 63.16% of the total analyzed samples, cannot be determined. The sensitivity observed in hair is lower than that previously reported (70%) for *Leishmania*-infected dogs [20]. This fact could be due to the fact that *Leishmania* causes skin disorders [10,39], and that the target gene used to detect *Leishmania* DNA was present in the order of 10,000–20,000 copies/parasite [40], whereas the target used in *Plasmodium* was present in a single copy/parasite. In our opinion we could improve the detection of *Plasmodium* DNA in hair using another target gene, for example the mitochondrial genome, considered to be present in a quantity from about 20 to 200 copies/parasite [8,13].

In addition, we believe that since malaria is a hematic parasitic infection, obtaining hair with bulbs could increase the sensitivity of the technique, as *Plasmodium* DNA could be incorporated into follicular cells by migration from the bloodstream, but this mechanism is still unclear. Therefore, further research taking this into account is needed to improve the sensitivity of the technique. The DNA presence seems to be strongly associated with the parasite load, since positive results belonged to patients with high amounts of DNA copies detected in blood and high blood thick smear parasitemia. However, the absence of DNA in hair samples could be due to the clinical stage (recently infected, chronic or clinically healed) of the patient at the moment of sampling. The sensitivity of the hair-qPCR method for malaria diagnosis must be the aim of wider molecular and clinical future studies as stated before. Despite the fact that the sensitivity of the hair-PCR method obtained in *Plasmodium* was too low to apply it as a medical diagnosis method, we think that it would have important implications in forensic science and also in paleoparasitology studies [41].

Despite the limitations of the study, mainly due to the sample size, we consider that this new finding has a notorious impact in clinical and care practice, not only for patients with malaria, but also for patients with other infectious diseases. Moreover, hair samples have numerous advantages. The collection is noninvasive, and transport and storage do not require special conditions.

Finally, we believe that countries with a greater socioeconomic level could collaborate in eradication of malaria becoming involved in the WHO universal access strategy for the malaria diagnosis.

## 5. Conclusions

*P. falciparum* DNA was successfully detected by qPCR in blood samples conserved in Whatman paper. After being shipped from Rwanda and submitted to several storage conditions, blood and plasmodial DNA remained stable, providing high sensitivity results in qPCR, compared to thick blood smear and RDT and confirming malaria diagnosis.

We must highlight that this study shows for the first time the presence of *P. falciparum* DNA in human hair detected by qPCR, supporting previous studies in animals infected with *Leishmania spp.* Our results might help to understand the role of hair and its physiological functions. Despite this, we cannot propose this method as a diagnostic tool until we conduct more studies, necessary to know the mechanism of the incorporation of DNA of *P. falciparum* in hair, which is unclear. However, we suggest that the findings revealed in our study could have several important practical applications. It is a noninvasive collection of samples, which avoid the suffer and the pain of the patient, transport and storage do not require special conditions, so it facilitates the universal access to the diagnosis and the detection of cases in the early stages of the disease.

## Figures and Tables

**Table 1:** Summary of clinical data, parasitological and immunological results of the 19 malaria patients.

No. Pat.	Age (Years)	Sex ^†^	Type of Malaria ^‡^	Treatment ^†††^	Parasite Load ^‡^	RDT ^§^/*		qPCR **	
						Ag Pf pHRP-II	Ag Pan pLDH	Blood	Hair
1	28	F	S	Quinine	++	Pos.	Neg.	Pos. (55.87)	Neg.
2	20	M	C	Quinine	++++	Pos.	Neg.	Pos. (2007.89)	Pos. (177.73)
3	22	M	C	Quinine	+	Neg.	Neg.	Pos. (14.74)	Neg.
4	24	M	U	Coartem	++	Pos.	Neg.	Pos. (312.47)	Neg.
5	28	F	U	Coartem	+	Pos.	Neg.	Pos. (11.68)	Neg.
6	4	F	C	None	++++	Pos.	Pos.	Pos. (1511.29)	Pos. (12.24)
7	17	F	U	None	++++	Pos.	Pos.	Pos. (818.04)	Pos. (20.17)
8	20	F	U	None	++++	Pos.	Pos.	Pos. (1280.01)	Pos. (9.26)
9	20	M	U	None	+++	Pos.	Pos.	Pos. (3580.96)	Pos. (6.68)
10	28	F	U	None	++++	Pos.	Pos.	Pos. (28976.48)	Neg.
11	25	F	C	Quinine	++++	Pos.	Pos.	Pos. (171040.16)	Pos. (73.77)
12	26	M	U	None	+	Pos.	Pos.	Pos. (14.02)	Neg.
13	29	M	U	None	++++	Pos.	Pos.	Pos. (59.76)	Neg.
14	31	F	S	Quinine	++++	Pos.	Pos.	Pos. (63595.54)	Neg.
15	22	F	S	None	++++	Pos.	Pos.	Pos. (15923.18)	Neg.
16	19	M	C	None	+++	Pos.	Pos.	Pos. (8913.24)	Pos. (3.8)
17	20	F	U	Coartem	++++	Pos.	Neg.	Pos. (1637.71)	Neg.
18	17	M	C	Quinine	++	Pos.	Neg.	Neg.	Neg.
19	21	M	S	None	++++	Pos.	Pos.	Pos. (1418.87)	Neg.
% Sensitivity (X positives/Y total samples analyzed)						94.74% (18/19)	63.16% (12/19)	94.74%(18/19)	36.84%(7/19)

^†^ F.: Female: M.: Male. ^‡^ Type of malaria: Uncomplicated (U); Severe (S) Cerebral (C). ^†††^ Parasite load: + = 1–10 parasites per 100 thick-film fields. ++ = 11–100 parasites per 100 thick-film fields +++ = 1–10 parasites per thick-film field ++++ = more than 10 parasites per thick-fil field ^§^ Rapid Diagnosis Test: Pf (pHRP-II) Ag detects *P. falciparum* infection; Pan (pLDH) Ag detects all malaria species. * Pos.: Positive; Neg.: Negative. ** Estimated quantity of copies of gene of *P. falciparum* detected per 50 µL of blood sample and approximately 25 cm of hair.
